# Role of Adipokines in Maternal Cardiac Function of Pregnancies Following Metabolic Bariatric Surgery

**DOI:** 10.1007/s11695-026-08581-w

**Published:** 2026-06-04

**Authors:** Maryam Rahmati Ahmadabadi, Deesha Patel, Brett Johnson, Alexander Dimitri Miras, Makrina Savvidou

**Affiliations:** 1 Department of Metabolism, Digestion and Reproduction, Imperial College London, London, UK; 2 Chelsea and Westminster Hospital, London, UK; 3 Department of Metabolism, Digestion and Reproduction, Imperial College London, London, UK; 4 Ulster University, Coleraine, UK

**Keywords:** Pregnancy after bariatric surgery, Obesity in pregnancy, Maternal cardiac function, Adipokines in pregnancy

## Abstract

**Objective:**

Maternal metabolic bariatric surgery is associated with improved maternal cardiovascular function and an altered adipokine (adiponectin and leptin) levels. The aim of this study was to investigate whether adipokine levels mediate the improvement in maternal cardiovascular function reported in pregnant women with previous metabolic bariatric surgery.

**Methods:**

The study included 41 pregnant women with previous metabolic bariatric surgery and 41 pregnant women without metabolic bariatric surgery but with similar early pregnancy body mass index (BMI). All participants underwent cardiac function assessment using two-dimensional echocardiography, at 11–14, 20–24 and 30–32 weeks of gestation. Maternal blood, collected at these three visits, was used for measurement of maternal adiponectin and leptin. Mixed-effects model analysis was used to assess the differences in adiponectin and leptin between groups, after adjustment for time, maternal age, ethnic group, parity, method of conception, BMI, smoking, gestational age at measurement and development of gestational diabetes mellitus. Pearson’s correlation was used to assess the correlation of maternal adiponectin and leptin levels with cardiac indices.

**Results:**

Overall, maternal adiponectin levels were higher in the post-metabolic bariatric surgery group, compared to the no surgery group (*p* = 0.008). However, leptin levels did not differ between groups (*p* = 0.76). Adiponectin levels were not associated with indices of maternal cardiac function including haemodynamics, cardiac geometry, diastolic, systolic and longitudinal function (*p* > 0.05). In contrast, maternal leptin levels positively correlated with maternal cardiac output in both the no surgery and post-metabolic bariatric surgery groups (*p* < 0.01 and *p* = 0.02, respectively).

**Conclusions:**

Although pre-pregnancy maternal metabolic bariatric surgery is associated with an improvement in maternal cardiac function, maternal adiponectin and leptin levels alone are unlikely to contribute to this improvement.

**Supplementary Information:**

The online version contains supplementary material available at 10.1007/s11695-026-08581-w.

## Introduction

Obesity rates worldwide have reached epidemic levels. Metabolic bariatric surgery is the most effective treatment for sustainable weight loss and has gained increasing popularity, particularly amongst women of childbearing age [1]. Several studies of cardiovascular function after metabolic bariatric surgery have reported reduced rates of hypertension and an improvement in the cardiac geometry and function [2]. Metabolic bariatric surgery induces significant weight loss but is also associated with several hormonal changes, including changes in adiponectin and leptin levels, that impact insulin resistance and may have cardiovascular effects [2–4]. Adipokines released from adipocytes are known to participate in molecular pathways that affect glucose metabolism as well as the heart via an “adipocardiac axis.” [5] They are modulated by metabolic bariatric surgery, which may therefore mediate some of their beneficial cardiac effects [5]. Hyperleptinemia has been shown to be cardioprotective and administration of leptin results in beneficial nitric oxide–mediated endothelial vasorelaxation [6], however, chronic hyperleptinemia can result in increased heart rate (HR), hypertrophy, intimal hyperplasia and heart failure [7]. Metabolic bariatric surgery is associated with a reduction in leptin levels which can be through both weight loss–dependent and independent mechanisms [5, 8]. Adiponectin has both anti-inflammatory and antidiabetic effects, though levels are decreased in obesity and diabetes [5, 8]. Low adiponectin concentrations are correlated with myocardial infarction, coronary atherosclerotic heart disease, hypertrophy, and hypertension [9, 10]. It has been demonstrated that adiponectin protects cardiovascular health through its vasodilatory, anti-apoptotic, anti-inflammatory and anti-oxidative activities [11, 12]. Studies have reported increased levels of adiponectin after metabolic bariatric surgery [8]. 

Healthy pregnancy is associated with significant maternal cardiovascular adaptation including increased cardiac output and left ventriclular remodeling [13]. Our previous work has demonstrated that pregnant women with previous metabolic bariatric surgery have more favorable haemodynamic variables, diastolic indices and cardiac geometry suggesting better cardiac health compared to pregnant women without previous metabolic bariatric surgery but similar early pregnancy or pre-surgery BMI [14, 15]. 

Considering that metabolic bariatric surgery is associated with altered circulating adipokine levels and that both adiponectin and leptin exhibit cardiovascular properties, it would be reasonable to hypothesise that these adipose-derived peptides may play a role in the regulation of maternal cardiovascular function. The aim of this study was to compare the maternal levels of adiponectin and leptin, during gestation, between pregnant women with and without previous metabolic bariatric surgery and investigate their role in maternal cardiac function, specifically blood pressure (BP), heart rate, cardiac output, relative wall thickness, left ventricular mass, mitral flow velocity (E/A ratio), tissue Doppler imaging at the lateral mitral annular velocity (E’) and mitral annular plan systolic excursion.

## Methods

This was a prospective, observational, longitudinal study conducted from April 2018 to June 2020 in an inner London teaching hospital, UK. The study included 41 pregnant women with previous metabolic bariatric surgery and 41 controls without surgery matched for early pregnancy BMI, age, ethnicity, and gestational diabetes mellitus status. The study protocol has been described previously; [14] briefly, pregnant women with previous metabolic bariatric surgery and those without were recruited from the antenatal clinic of our Hospital. Maternal demographic characteristics and medical history were recorded at the initial research visit. Women were seen at 11–14, 20–24 and 30–32 weeks of gestation, and at each visit, maternal weight, BMI and BP (measured in duplicate and mean value recorded) were measured and maternal blood samples were obtained. Maternal cardiac function was assessed at these three visits using transthoracic echocardiography, as previously described [14]. In brief, two-dimensional, M-mode, and tissue Doppler imaging (TDI) were performed using an Ie33 Philips Ultrasound system (Philips, Amsterdam, The Netherlands) according to European and American guidelines [16, 17]. The cardiac output (mL) was calculated as stroke volume X heart rate [18]. The stroke volume (mL) was calculated as the cross-sectional area of the left ventricular outflow tract velocity time integral [18]. The left ventricular mass (g) was calculated as (0.8 [1.04 ([interventricular septum diameter (mm) + left ventricle internal diameter (mm) + posterior wall thickness (mm)])^3^ -left ventricle internal diameter^3^ (mm)]) + 0.6g [16]. The relative wall thickness was calculated as (2 X posterior wall thickness [mm])/left ventricle internal diameter (mm) [16]. Diastolic function was assessed by mitral flow velocity (E/A ratio) and tissue Doppler imaging at the lateral mitral annular velocity (E’) [17]. The longitudinal function was assessed by mitral annular plane systolic excursion (MAPSE) at the lateral annulus [19]. 

Pregnancies were followed up until delivery and outcomes were recorded. Gestational age was calculated by crown-rump length at the dating scan (11–14 weeks). Gestational diabetes (GDM) was defined according to the National Institute of Health and Care Excellence (NICE) guidelines of fasting plasma glucose level ≥ 5.6 mmol/L and/or a 2- hour plasma glucose level ≥ 7.8 mmol/L [20]. Birthweight (BW) was recorded at birth and birthweight percentiles were calculated [21]. Pre-eclampsia was defined as new onset maternal BP ≥ 140/90 mmHg with proteinuria [22]. 

At the same three visits when echocardiography was performed (11–14, 20–24 and 30–32 weeks of gestation), stored maternal bloods were used for measurement of adiponectin and leptin (blood sample collection was at random times of the day). Maternal serum adiponectin and leptin levels were measured using ELISAs designed for human leptin and adiponectin (EZHL-80SK and EZHADP-61 K, Merck KGaA, Darmstadt, Germany). The samples were frozen, thawed and analysed once. The measurement process followed the manufacturer’s instructions, and the samples were measured in duplicate. The leptin assay intra- and inter-assay CV were 2.78% and 3.51%, lower limit of sensitivity was 0.78 ng/ml. The adiponectin assay intra- and inter-assay CV were 7.01% and 3.78%, lower limit of sensitivity was 0.2ng/ml. Kit quality control samples were included across all plates.

### Statistical analysis

Sample size calculation was performed using G*Power software (G*Power for Windows OS X, version 3.1; Heinrich-Heine‐Universität, Dusseldorf, Germany) [23]. There are no prior studies investigating the levels of adiponectin and leptin in pregnant women with previous metabolic bariatric surgery. Therefore it was difficult to estimate accurately the number of subjects required in each group to obtain results with adequate power. Using studies of individuals before and after metabolic bariatric surgery, outside the context of pregnancy, we estimated sample sizes for an alpha‐level of 0.05 and a power of 0.95. In order to detect a mean difference of 5.5 µg/ml in adiponectin, and 47.7ng/ml in leptin, a sample sizes of 14 would have been needed in each group [24]. 

The normality of the distribution was checked by the Kolmogorov-Smirnov test. Data were expressed as mean ± standard deviation or as median (interquartile range) for normally and not normally distributed data, respectively. Categorical data are expressed as n (%). Student’s t-test, Mann-Whitney or chi-square tests were used for the comparison of numerical and categorical data, respectively, between groups. Pearson’s correlation was used to assess the correlation of maternal adiponectin and leptin levels with the cardiac parameters measured. Hierarchical modelling was used for comparison of maternal adiponectin and leptin using multilevel linear mixed-effects models. The fixed effect component included time (three visits), study group (post-metabolic bariatric surgery, no-surgery), maternal age, ethnic group, parity, method of conception, BMI, smoking, gestational age at assessment, development of GDM and levels of adiponectin or leptin, when appropriate. Random effects were time and group. Estimated marginal means with 95% Confidence Intervals are given. Differences in the cardiac and adipokine levels were considered significant at *p* < 0.05, Bonferroni correction was implemented during the statistical analysis. All analyses were performed using IBM SPSS Statistics, 2022 (IBM Corp. Armonk, New York, USA) [25]. 

## Results

In this study a total of 41 pregnant women with previous metabolic bariatric surgery, and 41 controls were included. The analysis included women who attended a minimum of two out of the three research visits. Among the post-metabolic bariatric surgery women, 21 had undergone Roux-en-Y gastric bypass (RYGB), 13 had undergone vertical sleeve gastrectomy (VSG) and 7 had undergone gastric banding, the weight loss following each procedure was 37 kg, 30 kg and 20 kg respectively. The mean interval between the surgery and conception was 48.7 months. Although there were no significant differences between the groups, the post-metabolic bariatric surgery women delivered smaller babies compared to the no-surgery counterparts and were less likely to develop pre-eclampsia during pregnancy (Table 1).

**Table 1:** Patient characteristics of the study participants

Characteristics	Controls *N* = 41	Post-metabolic bariatric surgery *N* = 41	*P* value
Age (Years)	33.2 (5.2)	33.6 (5.7)	0.78
Ethnicity			
White n (%)	33 (80.5)	34 (82.9)	0.78
Other n (%)	8 (19.5)	7 (17.1)	
Parity			
Nulliparous n (%)	21(51.2)	23 (56.1)	0.66
Parous n (%)	20 (48.8)	18 (43.9)	
Body mass index at booking (kg/m^2^)	34.5 (6.8)	34.3 (7.1)	0.9
Conception			
Spontaneous, n (%)	35 (85.4)	39 (95.1)	0.14
Assisted, n (%)	6 (14.6)	2 (4.9)	
Smoking			
No n (%)	39 (95.1)	37 (90.2)	0.4
Yes n (%)	2 (4.9)	4 (9.8)	
Gestational diabetes mellitus			
No n (%)	34 (82.9)	34 (82.9)	1
Yes n (%)	7 (17.1)	7 (17.1)	
Pre-eclampsia			
No, n(%)	38 (92.7)	41(100)	0.06
Yes, n(%)	3 (7.3)	0	
Mode of delivery			
Vaginal delivery, n(%)	23 (56.1)	20 (48.8)	0.81
Caesarean delivery, n (%)	18 (43.9)	18 (43.9)	1
Gestational age at birth (weeks)	39.1(38.4–40.4)	38.8 (37.7–39.7)	0.34
Birth weight(gr)	3580 (3170–3805)	3110 (2530–3375)	**0.01**
Large for gestational age, n (%)	8 (19.5)	5 (12.2)	0.36
Small for gestational age, n (%)	2 (4.9)	12 (29.3)	**0.03**

Data is expressed as either mean (standard deviation), median (interquartile range), or n (percentage) (*p* < 0.05) [14]

**Fig. 1:**
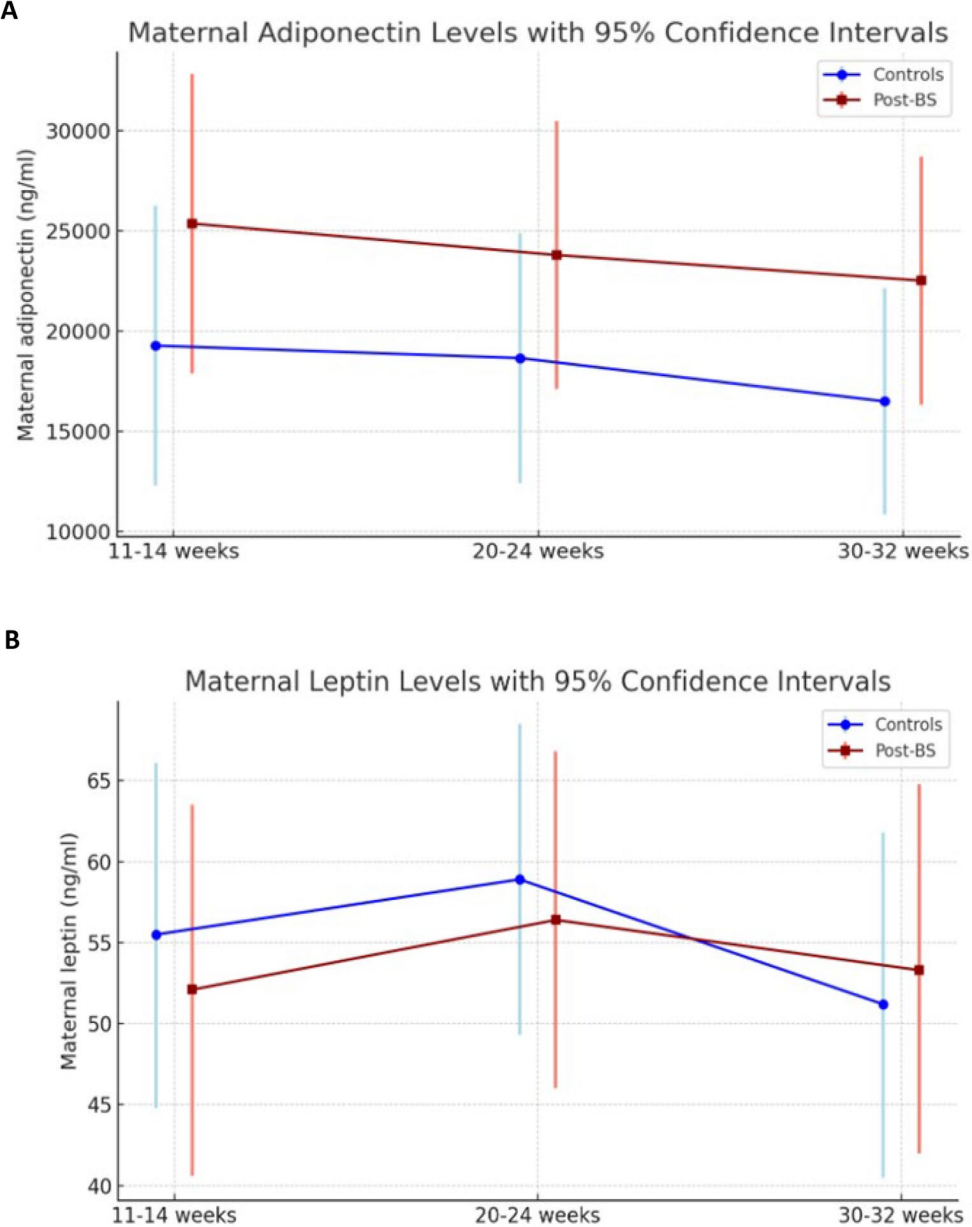
Multilevel linear mixed-effects model, estimated marginal means for maternal adiponectin and leptin levels across gestation in the no-surgery (controls) and post-metabolic bariatric surgery group (post-BS)

Mixed-effects model analysis was used compare maternal adiponectin and leptin levels between different groups at each trimester and overall (Fig. 1, supplementary Table 1). The analysis took into account various factors, including time (three visits), maternal age, ethnicity, parity, method of conception, BMI, smoking, gestational age at measurement, and the development of GDM. Overall across the trimesters, women with previous metabolic bariatric surgery had higher adiponectin levels when compared to controls (*p* = 0.008, effect size 0.49) but there were no differences in leptin levels between the groups (*p* = 0.76, effect size 0.09).

## Correlation between maternal adiponectin, leptin and cardiac indices

We investigated the association between maternal adiponectin and leptin levels with various cardiac parameters (Table 2) in the two groups of pregnant women across the three trimesters. There was no significant correlation between maternal adiponectin levels and cardiac indices. However, maternal leptin levels were found to have a positive correlation with maternal cardiac output and left ventricular mass. Using a mixed-effects model analysis and adjusting for maternal characteristics, we still found a positive association between maternal leptin levels and cardiac output, in both groups of pregnant women (*p* < 0.01 and *p* = 0.02 for the no-surgery and post-metabolic bariatric surgery group, respectively).

**Table 2:** Pearsons correlations between maternal adiponectin and leptin levels and cardiac indices at each trimester of pregnancy in the no-surgery group (A and B) and post-metabolic bariatric surgery group (C and D)

A) Correlations of maternal adiponectin in the no-surgery group						
	11-14 weeks		20-24 weeks		30-32 weeks	
	Correlation Coefficient	P value	Correlation Coefficient	P value	Correlation Coefficient	P value
SBP (mmHg)	-.287	0.146	-.163	0.308	.049	0.776
DBP (mmHg)	-.274	0.167	-.064	0.693	.240	0.159
HR (bpm)	-.428	0.026	-.293	0.063	-.143	0.404
CO (L/min)	-.282	0.155	.029	0.855	.034	0.845
RWT	-.069	0.739	-.116	0.476	-.145	0.405
LV mass (g)	-.116	0.574	-.217	0.178	-.022	0.899
E/A ratio	-.098	0.634	.236	0.137	.138	0.423
E’ lateral (m/s)	.092	0.654	.115	0.475	-.324	0.054
MAPSE L (mm)	.048	0.820	.179	0.274	.105	0.556
B) Correlations of maternal leptin levels in the no-surgery group						
	11-14 2weeks		20-24 weeks		30-32 weeks	
	Correlation Coefficient	P value	Correlation Coefficient	P value	Correlation Coefficient	P value
SBP (mmHg)	-.080	0.67	.039	0.808	.045	0.793
DBP (mmHg)	-.001	0.994	.037	0.819	.004	0.984
HR (bpm)	-.001	0.997	-.013	0.935	-.154	0.370
CO (l/min)	-.046	0.823	.024	0.879	.100	0.560
RWT	-.340	0.089	-.357	0.022	-.192	0.270
LV mass (g)	.150	0.463	-.042	0.792	-.235	0.174
E/A ratio	.237	0.255	.356	0.024	.292	0.084
E’ lateral (m/s)	.287	0.156	.068	0.671	.207	0.234
MAPSE L (mm)	-.059	0.785	.019	0.914	-.063	0.718
C) Correlations of maternal adiponectin levels in the postmetabolic bariatric surgery group						
	11-14 2weeks		20-24 weeks		30-32 weeks	
	Correlation Coefficient	P value	Correlation Coefficient	P value	Correlation Coefficient	P value
SBP (mmHg)	-.080	0.67	.039	0.808	.045	0.793
DBP (mmHg)	-.001	0.994	.037	0.819	.004	0.984
HR (bpm)	-.001	0.997	-.013	0.935	-.154	0.370
CO (l/min)	-.046	0.823	.024	0.879	.100	0.560
RWT	-.340	0.089	-.357	0.022	-.192	0.270
LV mass (g)	.150	0.463	-.042	0.792	-.235	0.174
E/A ratio	.237	0.255	.356	0.024	.292	0.084
E’ lateral (m/s)	.287	0.156	.068	0.671	.207	0.234
MAPSE L (mm)	-.059	0.785	.019	0.914	-.063	0.718
D) Correlations of maternal leptin levels in the post-metabolic bariatric surgery group						
	11-14 2weeks		20-24 weeks		30-32 weeks	
	Correlation Coefficient	P value	Correlation Coefficient	P value	Correlation Coefficient	P value
SBP (mmHg)	.420	0.033	.247	0.119	.301	0.074
DBP (mmHg)	.469	0.016	.297	0.060	.253	0.136
HR (bpm)	.127	0.537	.250	0.115	.344	0.040
CO (l/min)	.399	0.043	.481	0.001	.523	0.001
RWT	.009	0.965	.051	0.749	.056	0.747
LV mass (g)	.171	0.403	.262	0.099	.309	0.071
E/A ratio	-.020	0.925	-.287	0.073	.208	0.223
E’ lateral (m/s)	.033	0.873	-.128	0.426	-.194	0.263
MAPSE L (mm)	.028	0.898	-.141	0.413	.175	0.314

## Discussion

The study has found that pregnant women after metabolic bariatric surgery have significantly higher adiponectin levels compared to women that have not undergone surgery, but these elevated levels do not correlate with maternal cardiac function (including BP, cardiac output, E/A ratio and left ventricular mass). On the other hand, there is no notable disparity in maternal leptin levels between the groups and leptin levels appeared to correlate positively with maternal cardiac output but similarly in both groups. The above findings suggest that the improvement in maternal cardiovascular function previously observed in pregnant women with a history of metabolic bariatric surgery is unlikely to be mediated through adiponectin and leptin levels. Higher leptin typically reflects greater fat mass and may be associated with higher cardiac output due to increased metabolic demand or volume load [26]. 

In non-pregnant individuals, metabolic bariatric surgery has been shown to improve cardiovascular function including reduction in BP, epicardial fat, left ventricular mass and myocardial stiffness with enhanced performance during both systole and diastole [27]. The beneficial cardiovascular effects of metabolic bariatric surgery have been linked to significant weight loss and improvement in insulin sensitivity, along with other factors that have been implicated in these outcomes [28]. Metabolic bariatric surgery has been shown to result in elevated adiponectin levels and decreased leptin levels [8]. Adiponectin has vasodilatory effects, promoting vascular health and preventing hypertension and atherosclerosis [10]. In contrast, leptin can have negative effects on cardiovascular health as high levels are linked to increased inflammation and oxidative stress, which can contribute to cardiac remodelling, increased BP, heart rate and possibly, cardiac hypertrophy and cardiovascular disease [29]. Considering the role of these adipokines in the circulatory system, it has been hypothesised that the changes in their level following metabolic bariatric surgery could contribute to the observed improvement in cardiovascular function, seen following metabolic bariatric surgery. However, no previous study examined the direct association between adiponectin/leptin levels and parameters of cardiovascular performance in post-metabolic bariatric surgery individuals.

Our previous work has demonstrated that, compared to pregnant women without previous metabolic bariatric surgery, those with surgery have shown improved hemodynamics, including lower BP, heart rate, cardiac output and better cardiac diastolic function, indicated by a higher E/A ratio and tissue Doppler imaging E’ at the lateral mitral annulus [14]. However, in the current study we did not find a correlation between these improved cardiac indices and maternal adiponectin levels, which, however, were found to be higher in this group of pregnant women; in accordance with studies outside pregnancy [30]. Based on these findings, maternal adiponectin is unlikely to play a key role in regulating maternal cardiovascular function in pregnancies after metabolic bariatric surgery.

It has also been proposed that decreased leptin levels, seen after metabolic bariatric surgery outside of the setting of pregnancy, may play a role in reducing left ventricular mass and improving diastolic and aortic elastic function [28]. In the current study, we demonstrate a significant positive association between leptin levels and certain cardiac parameters including cardiac output and left ventricular mass (Table 2). Nevertheless, it is noteworthy that these associations were comparable in both the group of pregnant women with and without metabolic bariatric surgery. Furthermore, there were no notable disparities in leptin levels observed between the two groups, potentially due to similar BMI in both groups. These findings suggest that leptin alone is unlikely to account for the observed cardiovascular differences described in our study.

Based on these findings, it is likely that other factors, such as improvement in insulin resistance, changes in the autonomic nervous system, weight loss, etc., beyond leptin levels, contribute to the enhanced cardiovascular function observed in pregnant women with previous metabolic bariatric surgery. These potential mechanisms are discussed in following paragraphs.

Metabolic bariatric surgery is associated with considerable metabolic changes and weight loss. Significant weight loss can directly impact cardiovascular function by reducing body weight and adiposity, which can alleviate stress on the cardiovascular system and enhance cardiac indices [5]. In addition, there is improvements in insulin sensitivity [31] which has also been demonstrated in pregnancy following metabolic bariatric surgery [32]. Obesity is assoicaited with persistent low-level inflammation and metabolic bariatric surgery has been shown to decrease systemic inflammatory markers like C-reactive protein, leucocytes and ferritin; this reduction in inflammation factors may potentially have a positive impact on the cardiovascular system [33]. 

Furthermore, metabolic bariatric surgery results in changes in gut hormone levels such as ghrelin, cholecystokinin (CCK) and resistin and through the entero-cardiac axis has a positive impact on cardiovascular function [34, 35]. Specifically, it is reported that ghrelin may contribute to lowering BP and heart rate, improvement in lipid profile and better glycemic control. Additionally, it has been found to have vasodilatory effects, which may help to lower BP [36]. Resistin has been shown to play a role in atherogenesis and is associated with vascular inflammation, lipid accumulation, and plaque vulnerability; the decreased resistin levels after metabolic bariatric surgery may contribut to a reduction in post-surgery cardiac events [5, 37]. The primary role of CCK is related to digestion and satiety, however, there is some evidence suggesting its potential impact on cardiovascular health [38, 39]. Increased CCK levels may contribute to post-surgical changes in appetite regulation and satiety, which can aid in weight loss and metabolic improvements [39]. Metabolic bariatric surgery has been associated with a decrease in sympathetic nervous system activity and amelioration of the sympathetic baroreflex activity which could contribute to the differences in haemodynamic indices, such as heart rate, BP and overall cardiac function, observed between our no-surgery and post-metabolic bariatric surgery pregnant groups [40]. It is worth mentioning that the interplay between multiple factors can influence maternal cardiovascular function.

### Strengths and limitations

This prospective, longitudinal study included participants from varied demographic backgrounds, increasing the external validity of the findings. The two groups were well matched regarding maternal characteristics and in particular early pregnancy BMI. All maternal cardiac assessments were performed by experienced operators, with reliable inter- and intra-observer variability, and have been published before [14]. Measurements of adiponectin and leptin were performed in duplicates, using a reliable technique, in order to reduce variations. The use of multilevel linear mixed-effects model analysis enabled adjustment for possible confounders and provided a comprehensive understanding of the relationships between variables. However, the participants were recruited from a single medical centre which may restrict the generalizability of the findings to other healthcare settings or regions. We included a relatively small number of cases but the longitudinal design of the study adds strength to our findings, additionally, the power calculation although used studies outside of pregnancy, supported our sample size. However, we recognise that for the correlation between adipokines and cardiac inidices, a larger sample size may have yielded further results. We acknowledge we were not able to assess the different types of surgery due to the relative rarity of metabolic bariatric surgery in pregnancy or lifestyle differences such as physical activity and diet. Levels of adiponectin and leptin were not measured in a fasted state, however, as this was the case for both groups it is unlikely to influence the findings. In addition, metabolic factors (insulin resistance markers or inflammatory cytokines) were not measured and our study was limited to adipokines, these biomarkers would be appropriate for future work.

To conclude, this study found that women with previous metabolic bariatric surgery have, overall, higher adiponectin levels compared to pregnant women without previous metabolic bariatric surgery, but the leptin levels are similar between the groups. Maternal adiponectin levels did not, whereas leptin levels did correlate with some indices of maternal cardiovascular function but this correlation was similar in the two groups. These results suggest that maternal adiponectin and leptin levels do not directly mediate the improvement in maternal cardiovascular function observed after metabolic bariatric surgery. Further research is required to investigate the mechanisms and factors that may influence maternal cardiac performance.

## Supplementary Information

Below is the link to the electronic supplementary material.

Supplementary Material 1

## Data Availability

Data is provided within the manuscript, further data is available on request.
