# Supplementary material for: Role of Adipokines in Maternal Cardiac Function of Pregnancies Following Metabolic Bariatric Surgery
Source: Obes Surg. 2026 Jun 4;36(7):3558–65. doi: 10.1007/s11695-026-08581-w (PMC13323739; doi:10.1007/s11695-026-08581-w)
Supplement: Supplementary file 1 — Supplementary Material 1 [file 11695_2026_8581_MOESM1_ESM.docx]

**Supplementary Table 1.** Multilevel linear mixed-effects models for maternal adiponectin (A) and leptin (B) levels. Estimated marginal means given for each trimester and group with 95% confidence interval

1. **Adiponectin (ng/ml)**

|  | **Controls** | **Post-metabolic bariatric surgery** | **P value** |
| --- | --- | --- | --- |
| 11-14 weeks | 19262.9 (12263.8 - 26262.1) | 25354.4 (17892.8 - 32815.9) | 0.14 |
| 20-24 weeks | 18642.5 (12407.2 - 24877.8) | 23786.2 (17110.2 - 30462.1) | 0.10 |
| 30-32 weeks | 16481.2 (10841.6 - 22120.7) | 22506.7 (16324.1 - 28689.3) | 0.05 |
|  | | | |
| Overall mean difference | | 5753.5 | 0.008 |
| Effect size Cohen’s d | | 0.49 | |

1. **Leptin (ng/ml)**

|  | **Controls** | **Post-metabolic bariatric surgery** | **P value** |
| --- | --- | --- | --- |
| 11-14 weeks | 55.5 (44.8 - 66.1) | 52.1 (40.6 - 63.5) | 0.26 |
| 20-24 weeks | 58.9 (49.3 - 68.5) | 56.4 (46.0 - 66.8) | 0.23 |
| 30-32 weeks | 51.2 (40.5 - 61.8) | 53.3 (42.0 - 64.8) | 0.97 |
|  | | | |
| Overall mean difference | | 1.2 | 0.76 |
| Effect size Cohen’s d | | 0.09 | |
